# Poor methodological detail precludes experimental repeatability and hampers synthesis in ecology

**DOI:** 10.1002/ece3.1722

**Published:** 2015-09-23

**Authors:** Neal R. Haddaway, Jos T.A. Verhoeven

**Affiliations:** ^1^ MISTRA EviEMRoyal Swedish Academy of Sciences114 18StockholmSweden; ^2^ Ecology and BiodiversityDepartment of BiologyUtrecht UniversityPadualaan 83584CH UtrechtThe Netherlands

**Keywords:** Evidence synthesis, experimental design, meta‐analysis, reliability, research legacy, susceptibility to bias, systematic review, transparency

## Abstract

Despite the scientific method's central tenets of reproducibility (the ability to obtain similar results when repeated) and repeatability (the ability to replicate an experiment based on methods described), published ecological research continues to fail to provide sufficient methodological detail to allow either repeatability of verification. Recent systematic reviews highlight the problem, with one example demonstrating that an average of 13% of studies per year (±8.0 [SD]) failed to report sample sizes. The problem affects the ability to verify the accuracy of any analysis, to repeat methods used, and to assimilate the study findings into powerful and useful meta‐analyses. The problem is common in a variety of ecological topics examined to date, and despite previous calls for improved reporting and metadata archiving, which could indirectly alleviate the problem, there is no indication of an improvement in reporting standards over time. Here, we call on authors, editors, and peer reviewers to consider repeatability as a top priority when evaluating research manuscripts, bearing in mind that legacy and integration into the evidence base can drastically improve the impact of individual research reports.

## The Problem

A central tenet in scientific research is that theories should be testable and refutable (Popper 1968) and experiments that test these theories should be repeatable (Gurevitch et al. 2001; Koricheva 2003). Research repeatability through transparent description of study design and methodology is paramount to ensuring reliability of study findings. Related to this, reproducibility refers to the ability to obtain a similar finding when repeating a method (Slezák and Waczulíková 2011). Despite this universally accepted logic, we have found that poor methodological detail in published ecological research is common and threatens its robustness, impact, and legacy.

Critical appraisal is a key requirement of *systematic reviews*; robust approaches to reviewing existing research evidence using strict methods set out by review coordinating bodies, such as the Cochrane Collaboration (www.cochrane.org) or the Collaboration for Environmental Evidence (www.environmentalevidence.org). The opportunities for reaching new overall conclusions on pressing fundamental and applied research questions have grown considerably with the availability of new statistical approaches for meta‐analysis. However, through our experience of critical appraisal of large bodies of evidence, we commonly find published academic research articles that do not provide sufficient methodological detail for studies to be repeated. For example, a systematic map of the environmental and socioeconomic impacts of high altitude land abandonment identified 111 of 190 studies as being described with low methodological detail (Haddaway et al. 2014a). More specifically, 38 studies failed to report the timing of investigation, 40 studies failed to report intervention duration, 28 studies failed to describe the degree of replication, and 105 studies did not describe the spatial scale over which experiments took place. Similarly, a systematic review of the impacts of land management on greenhouse gas and carbon flux in boreo‐temperate lowland peatlands found 39 of 140 studies to have poor methodological detail: for example, not stating the timescale of management activities, the period or timing of sampling, giving no indication of the number of replicates used, and failing to describe the relative locations of control and treatment areas (Haddaway et al. 2014b). Two similar systematic reviews of the quantitative removal of total nitrogen and phosphorus from water flowing through constructed wetlands in the British Isles (Palmer‐Felgate et al. 2013) and globally (Land et al. 2013) encountered poor methodological detail. In one of these reviews, 67 of 121 studies provided insufficient methodological detail and/or statistical design to allow for meaningful synthesis (Land et al. 2013). In a final example, an ongoing systematic review of the impacts of farmland management on soil organic carbon (Söderström et al. 2014), 70 of 500 studies failed to state their experimental design (i.e., split plot, randomized block).

**Figure 1:**
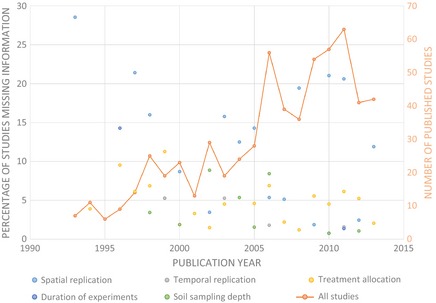
Missing information in study methods (expressed as the percentage of total studies published in each year) precluding replication across five domains for studies included in a systematic review of the impacts of agricultural management on soil organic carbon (unpublished data based on data from an ongoing systematic review according to Söderström et al. 2014, currently in review). Also displayed are the total number of studies published in each year (secondary *y*‐axis).

These problems are not simply restricted to systematic reviews: Similar problems with missing information occur in meta‐analyses (e.g., Garssen et al. 2014), which are a widely used synthesis tool in ecology (Stewart 2010). Previous authors have raised similar concerns over the need to make primary data available (Whitlock 2011) and that this should be accompanied by clear metadata (Michener et al. 1997). One recent systematic review of the impacts of agricultural land management on soil organic carbon found missing data to remain a significant problem over the last 22 years, with an average of 13% of studies per year (±8.0 [SD]) failing to report sample size, for example (Fig. 1). Many journals have responded positively and now require data to be archived alongside primary research articles. These calls relate to analytical reproducibility (the ability to reach the same conclusions) and not experimental repeatability (the ability to repeat the experiment described). Without explicit details of experimental design, the science behind the study cannot be repeated and study results cannot be synthesized.

Improving the availability of primary data is not an adequate solution for the problem of inadequate methodological detail. While missing quantitative data (such as means and standard deviations) prevent reliable studies from being included in statistical meta‐analysis (Haddaway 2015), missing methodological data prevent a judgement concerning reliability of the study from being made. Methods are available that allow studies missing quantitative data to be included to varying degrees in meta‐analyses, typically referred to as imputation (e.g., Furukawa et al. 2006). Even the studies that cannot be included through imputation “count” in syntheses because their existence is noted and their results can be discussed in a narrative. Studies that lack critical methodological detail, however, should not be included because their results may be unreliable. The relative risk of this unreliability depends upon the type of information missing, and “gap filling” methods can, in part, help reviewers (see Potential Solutions, below).

## Potential Solutions

Several solutions exist where primary research is missing methodological information:

1. contact corresponding authors with requests for information
2. check whether related manuscripts have been published for the same experiment and extract methodological details where methods can be reliably assumed to be the same
3. perform sensitivity analysis in meta‐analysis to examine the influence of studies missing vital methodological information (but that also provide sufficient quantitative data)
4. once found, publish missing information in a dedicated database (e.g., SRDR [http://srdr.ahrq.gov] or postpublication platform such as PubPeer (https://pubpeer.com) making it easier for future readers to find
5. promote improved reporting standards in the long term
  - promote current journal guidelines (e.g., Hillebrand and Gurevitch 2013) and establish universal mandates for methodological detail
  - improve instructions to peer reviewers to ensure they screen manuscripts for methodological repeatability
  - increase awareness of the importance of repeatability, particularly with respect to secondary synthesis and its benefits to legacy and impact.

The first three options are broadly suitable and require minimal effort. However, response rates for email addresses that are older than 3 to 5 years may be expected to be particularly low given movement of researchers between institutions. Options 4 and 5 may require considerable effort and require collective effort by the scientific community.

Similar concerns regarding missing methodological information have been raised in other disciplines (Altman 2015) and in relation to missing quantitative data that preclude further synthesis (Lajeunesse and Forbes 2003; Hillebrand and Gurevitch 2013; Ellington et al. 2015). We echo these calls by encouraging the research community to ensure that research is described in a way that is truly repeatable. Based on our experiences of critical appraisal in systematic reviews, we recommend the following minimum requirements be observed for manuscripts documenting experimental and quasi‐experimental studies:

1 experimental setting
  - field studies: detailed study location (latitude and longitude), influential climatic conditions
  - laboratory studies: controlled conditions (temperature, light cycle, influential reagents)
2 study date(s) and duration
3 selection procedures for sample selection and treatment allocation (purposeful, randomization, blocking, etc.)
4 level of true replication
5 level of subsampling (number and repeat or within‐replicate sampling)
6 sampling precision (within‐replicate sampling or pseudoreplication^1^)
7 study spatial scale (size of replicates *and* spatial scale of study area)
8 study design (i.e., before–after, control–impacts, time series, before–after‐control–impacts)
9 outcome measurement methods and equipment
10 description of any data manipulation, modeling, or statistical analysis undertaken

These are not onerous requirements, and despite being the subject of previous calls to adequately document archived data, we must reiterate the need for this information again to ensure legacy of primary research is maximized. We also advocate calls for better reporting of summary data (i.e., means, variability, and sample sizes) that permit meta‐analysis (e.g., Haddaway 2015), a valuable method in synthesizing results from multiple studies (Stewart 2010). Inclusion of these details will ensure study results are truly verifiable and have a legacy and impact beyond acting as a case study. As this information is readily available to authors, its required inclusion should be clearly specified in the “guide for authors” of peer‐reviewed journals and checked by journal referees. If a more strict code of conduct could become common practice in scientific reporting, the feasibility and success of large meta‐analyses and systematic reviews would be greatly enhanced.

## Conflict of Interest

None declared.

## Note

1 Pseudoreplication occurs where multiple samples are taken from within a true replicate (i.e., there is a lack of statistical independence) (Hurlbert 1984). Such sampling is useful when treated appropriately (Oksanen 2001) and can be used to indicate measurement precision or the accuracy of repeated measures but cannot be substituted for true replication needed for statistical analysis of variance.
